# Supplementary material for: Good performance of turquoise killifish (Nothobranchius furzeri) on pelleted diet as a step towards husbandry standardization
Source: Sci Rep. 2020 Jun 2;10:8986. doi: 10.1038/s41598-020-65930-0 (PMC7265286; doi:10.1038/s41598-020-65930-0)
Supplement: Supplementary file 1 — Supplementary Information. [file 41598_2020_65930_MOESM1_ESM.pdf]

SCIENTIFIC REPORTS

SUPPLEMENTARY MATERIALS

Good performance of turquoise killifish (*Nothobranchius furzeri*) on pelleted diet as a step towards husbandry standardization

Jakub Žák[1,2], Iva Dyková[3], Martin Reichard[1]*

[1] Czech Academy of Sciences, Institute of Vertebrate Biology, Květná 8, 603 65, Brno, Czech Republic

[2] Department of Zoology, Faculty of Sciences, Charles University, Viničná 7, 122 44, Prague, Czech Republic

[3] Department of Botany and Zoology, Faculty of Science, Masaryk University, Kotlářská 2, Brno, 611 37 Czech Republic

KILLIFISH HOUSING

All experimental work was completed on the wild-derived strain MZCS 222 [1] of turquoise killifish (*Nothobranchius furzeri*) bred at the accredited breeding facility at the Institute of Vertebrate Biology from 26 March to 21 May 2019. Fish were hatched and raised in common tanks until the age of 12 days following Polačik et al [2]. At the age of 12 days, fish were moved to six 35L glass tanks (30 fish per tank, three replicates for the bloodworm treatment and three replicates for pellets). The light regime was 14L:10D.

Fish were sorted by size at the age of 15, 17 and 20 days to reduce the negative effects of aggression and fish density on growth [2,3] (Supplementary Table S1). At the age of 29 days, final experimental groups (four replicates per treatment, 4 males + 8 females per 35L tank) were established. A female-biased sex ratio was chosen to reduce female harassment and male-male aggression [2]. Keeping fish in social groups improves fish willingness to feed and therefore promotes easier recognition of new food items [4]. Any dead or belly-sliding (common in captive *Nothobranchius* spp. [5] fish) were replaced by a similarly-sized individual from an additional tank with an identical setup to the experimental tanks. The number of replaced fish between dietary groups was similar (12 in the bloodworm treatment and 8 in the pellet treatment). The experiment was terminated when fish reached asymptotic growth at the age of 56 days.

WATER QUALITY

Nothobranchius furzeri is exceptionally tolerant species to water quality[2] because it inhabits ephemeral savanna pools with variable water quality and frequent presence of cattle dung in pools [6]. It has also higher tolerance to organic and inorganic toxic compounds than established toxicological models [7]. To sustain ideal water quality, each tank was equipped with an air-powered 15×15 cm sponge filter (EasyFish XY-2837, www.easyfish.cz). Manufacturer

states that this size of sponge filter is designed for tanks up to 300L which supports effective biofiltration in our experimental setup (35L tanks) and ensures stable water parameters in all tanks (Supplementary Table S2). Throughout experiment, 1/3 of water volume was changed every 2-3 days by tap water. Water temperature was maintained at 27.1 °C ± 0.87 (mean ± SD, recorded at 1 hour interval by two HOBO UA-002–64 loggers, Onset Computer, Bourne, MA, USA). When high amount of suspended material was found in bottom of aquaria and/or slight turbidity of water appeared (likely caused by dissolved colorants from bloodworms because water parameters did not differ), suspended material was siphoned out and additional water exchange was done.

To have a quantitative measure of water parameters, we have replicated our dietetic experiment (fish hatched 9 Dec 2019, water parameters measured 26 Feb 2020) with identical experimental setup and conducted additional quantitative measurements. Water conductivity was measured by waterproof conductometer Hanna HI 98312 (Hanna instruments, USA, www.hannainst.com). Dissolved oxygen was measured by YSI ProODO hand-held meter (YSI Inc./Xylem Inc, USA, www.ysi.com). Total ammonia nitrogen (TAN) was estimated by AM-test (Aquar, Czech Republic, www.aquar.cz). Water parameters such as NO3, NO2, total water hardness (GH), carbonate water hardness (KH) and pH were estimated by eSHa Aquatest (eSHa labs, Netherlands, www.eshalabs.eu). To ensure appropriate measurements, we have measured water parameters in empty aquaria as a negative control and in 300 liter aquaria with 25 cichlids (*Ctenochromis horei*) fed by Biomar Inicio 1.5 mm as a positive control. Water parameters in killifish tanks were in normal range recommended for this species [2] and did not differed between dietary groups (Supplementary Table 2).

SUPPLEMENTARY FIGURES AND TABLES

Figure S1

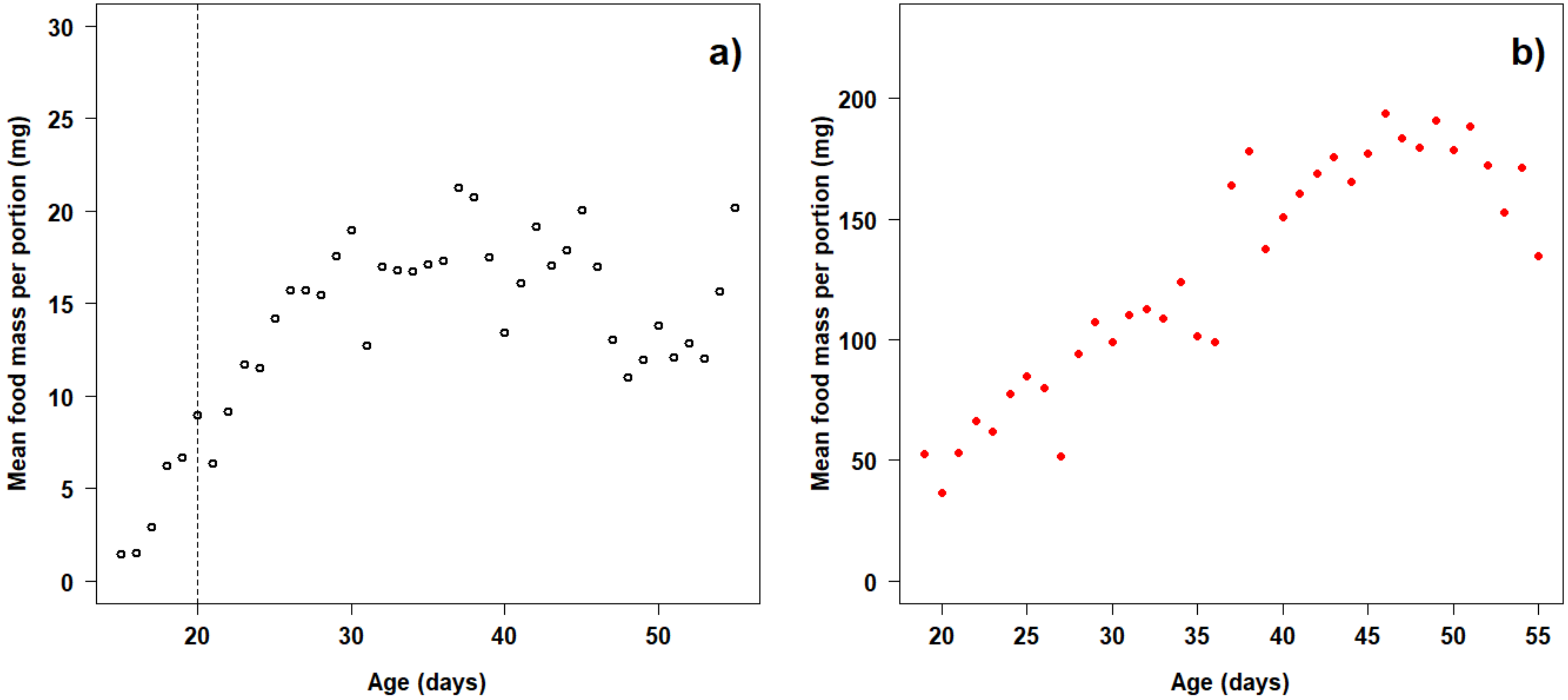

Figure S1: Mean food mass consumed by one killifish during one feeding event. Values are the mean from all feeding events on a given day. Note the different y-axis scale for each diet. Dashed line is age when all fish were adapted to pellets. a) pellets, b) bloodworms

Figure S2

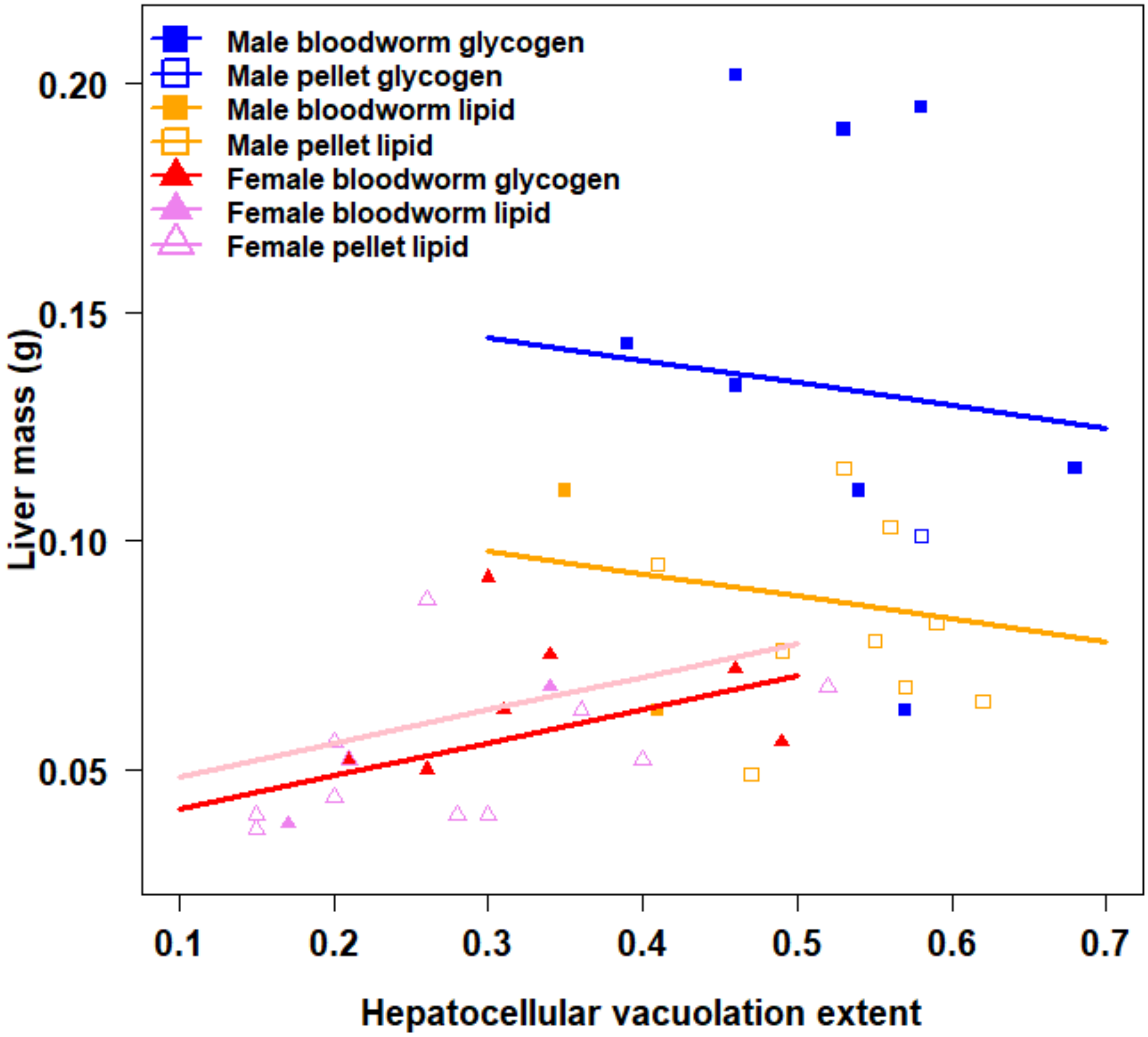

Figure S2: Relationship between liver mass and extent of hepatocellular vacuolation. Liver mass in females increases with the extent of hepatocellular vacuolation which was not observed in males. Points of observation represent raw data. Lines are simple trends from linear relationships.

Figure S3

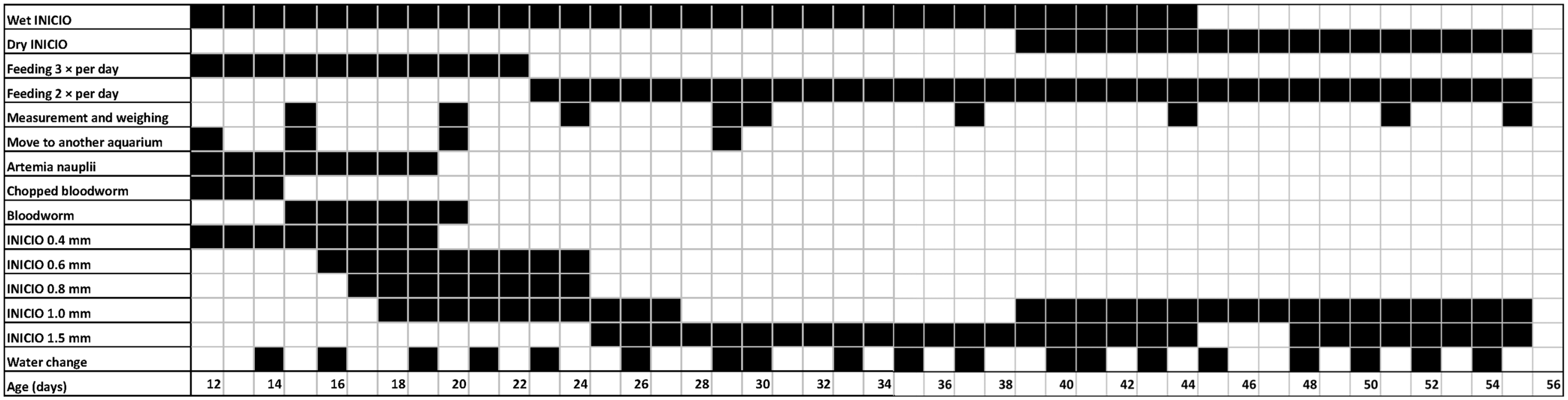

Figure S3: Schematic description of feeding regime (formulated diet BioMar INICIO) and killifish husbandry. Black cell stands for activity performed. See chapters "Killifish origin and husbandry" and "Killifish feeding procedures" for details.

Table S1: Overview of fish density during the experiment. During the juvenile period regular size assortment of fish was needed to achieve balanced growth. INICIO stands for BioMar starter food INICIO and number behind is for the pellet size fed to killifish. Experimental group "B" stands for bloodworm dietary group and "P" stands for pellets. Sex ratio was determined at the end of juvenile period before assembling final experimental tanks. Killifish size group is a result of regular fish assortment; similarly sized individuals were released together to have equal chance to acquire food. Small: the smallest individuals, Average: medium sized individuals, Large: largest individuals.

Age (days)	Tank volume (L)	Number of tanks	Experimental group	Diet	Number of fish per tank	Sex ratio M:F	Fish size group
0-3	6	1	-	*Artemia naupli*	Cca 200	J	All
3-12	75	2	-	*Artemia naupli*	Cca 90	J	All
13-14	35	3	B	Chopped bloodworms + *Artemia naupli*	30	J	All
13-14	35	3	P	Chopped bloodworms + *Artemia naupli*	30	J	All
15-16	35	3	B	Bloodworms + *Artemia naupli*	25	J	Average
15-16	35	3	P	Bloodworms + INICIO 0.4 + *Artemia naupli*	25	J	Average
15-16	35	1	B	Bloodworms	15	J	Large
15-16	35	1	P	Bloodworms + INICIO 0.4	15	J	Large
17-19	35	1	B	Bloodworms + *Artemia naupli*	10	J	Small
17-19	35	1	P	INICIO 0.4 + Bloodworms + *Artemia naupli*	11	J	Small
17-19	35	1	B	Bloodworms	15	J	Large
17-19	35	1	P	INICIO 0.8 +INICIO 1	15	J	Large
17-19	35	3	B	Bloodworms	20-21	J	Average
17-19	35	3	P	Bloodworms + INICIO 0.4 + INICIO 0.6	20-21	J	Average
20-28	35	3	B	Bloodworms	15-17	14:32	Average
20-28	35	3	P	INICIO 0.8 + INICIO 1	15-17	18:30	Average
20-28	35	2	B	Bloodworms	11 & 14	20:5	Large
20-28	35	2	P	INICIO 1	12 & 16	25:3	Large
20-28	35	1	B	Bloodworms	9	7:2	Small
20-28	35	1	P	INICIO 0.6	10	6:4	Small
29-56	35	4	B	Bloodworms	12	4:8	All
29-56	35	4	P	INICIO 1 + INICIO 1.5	12	4:8	All

Supplementary Table S2: Quantitative water parameters in replicated experiment. TAN stands for Total ammonia nitrogen. Control tank with *Ctenochromis horei* was fed by Biomar Inicio 1.5 mm.

Tank	diet	µS	Oxygen concentration mg×l^{-1}	Oxygen saturation (%)	pH	GH (°dH)	TAN (mg×l^{-1})	KH (°dH)	NO_2	NO_3	Cl
1	P	440	7.2	82	7.4	7-14	< 0.3	10	undetectable	25	undetectable
2	B	450	7.2	82	7.2	7-14	< 0.3	10	undetectable	25	undetectable
3	P	450	7.3	82	7.2	7-14	< 0.3	10	undetectable	25	undetectable
4	B	450	7.2	82	7.1	7-14	< 0.3	10	undetectable	25	undetectable
5	B	460	7.2	81	7.2	7-14	0.0	10	undetectable	25	undetectable
6	P	450	7.3	82	7.1	7-14	< 0.3	10	undetectable	25	undetectable
7	B	450	7.3	82	7.1	7-14	< 0.3	10	undetectable	25	undetectable
8	P	450	7.4	82	7.1	7-14	< 0.3	10	undetectable	25	undetectable
9	B	460	7.3	81	7.0	7-14	0.0	10	undetectable	25	undetectable
10	P	450	7.4	81	7.1	7-14	< 0.3	10	undetectable	25	undetectable
11	B	450	7.4	82	7.1	7-14	< 0.3	10	undetectable	25	undetectable
12	P	470	7.1	80	7.1	7-14	< 0.3	10	undetectable	25	undetectable

Control measurements

Tank	diet	µS	Oxygen concentration mg×l^{-1}	Oxygen saturation (%)	pH	GH (°dH)	TAN (mg×l^{-1})	KH (°dH)	NO_2	NO_3	Cl
Clear 35 L tank	-	330	7.0	80	6.9	7-14	0.0	8	undetectable	10	undetectable
300 L tank with 25 *C. horei*	P	530	6.9	80	7.0	7-14	< 0.3	8	undetectable	65	undetectable

REFERENCE:

1. Cellerino, A., Valenzano, D. R. & Reichard, M. From the bush to the bench: The annual Nothobranchius fishes as a new model system in biology. *Biol. Rev.* **91**, 511–533 (2016).

2. Polačik, M., Blažek, R. & Reichard, M. Laboratory breeding of the short-lived annual killifish Nothobranchius furzeri. *Nat. Protoc.* **11**, 1396–1413 (2016).

3. Vrtílek, M., Žák, J., Polačik, M., Blažek, R. & Reichard, M. Rapid growth and large body size in annual fish populations are compromised by density-dependent regulation. *J. Fish Biol.* (2019) doi:https://doi.org/10.1111/jfb.14052.

4. Lepič, P., Buřič, M. & Kozák, P. Adaptation to pelleted feed in pikeperch fingerlings: learning from the trainer fish over gradual adaptation from natural food. *Aquat. Living Resour.* **30**, (2017).

5. Dyková, I., Blažek, R., Součková, K., Reichard, M. & Slabý, O. Spontaneous adenocarcinoma of the gas gland in Nothobranchius fishes. *Dis. Aquat. Organ.* (2020) doi:10.3354/dao03437.

6. Reichard, M., Polačik, M. & Sedláček, O. Distribution, colour polymorphism and habitat use of the African killifish *Nothobranchius furzeri*, the vertebrate with the shortest life span. *J. Fish Biol.* **74**, 198–212 (2009).

7. Philippe, C. *et al.* Protocol for acute and chronic ecotoxicity testing of the turquoise Killifish Nothobranchius Furzeri. *J. Vis. Exp.* e57308 (2018) doi:10.3791/57308.
